# Activated Carbon/ZnFe_2_O_4_ Nanocomposite Adsorbent for Efficient Removal of Crystal Violet Cationic Dye from Aqueous Solutions

**DOI:** 10.3390/nano12183224

**Published:** 2022-09-16

**Authors:** Tahani Saad Algarni, Amal M. Al-Mohaimeed, Abdel-Basit Al-Odayni, Naaser A. Y. Abduh

**Affiliations:** 1 Department of Chemistry, College of Science, King Saud University, Riyadh 11451, Saudi Arabia; 2 Restorative Dental Sciences Department, College of Dentistry, King Saud University, Riyadh 11545, Saudi Arabia

**Keywords:** ZnFe_2_O_4_ nanocomposite, activated carbon, crystal violet, adsorption, water treatment

## Abstract

The aim of this study was to investigate the potential advantage of ZnFe_2_O_4_-incorporated activated carbon (ZFAC), fabricated via a simple wet homogenization, on the removal of cationic dye crystal violet (CV) from its aqueous solutions. The as-prepared ZFAC nanocomposite was characterized using Fourier transform infrared (FTIR), X-ray diffraction (XRD), nitrogen adsorption, scanning electron microscope (SEM), thermogravimetric analysis (TGA), and ultraviolet–visible (UV–Vis). Batch adsorption operating conditions such as the pH (3–11), CV concentration (25–200 ppm), ZFAC dose (10–50 mg), temperature (23–45 °C), and contact time were evaluated. The results indicate pH-dependent uptake (optimum at pH 7.2) increased with temperature and CV concentration increase and decreased as adsorbent dose increased. Modeling of experimental data revealed better fit to the Langmuir than Freundlich and Temkin isotherms, with maximum monolayer capacities (*Q_m_*) of 208.29, 234.03, and 246.19 mg/g at 23, 35, and 45 °C, respectively. Kinetic studies suggest pseudo-second order; however, the intra-particle diffusion model indicates a rate-limiting step controlled by film diffusion mechanism. Based on the thermodynamic parameters, the sorption is spontaneous (−Δ*G*°), endothermic (+Δ*H*°), and random process (+Δ*S*°), and their values support the physical adsorption mechanism. In addition to the ease of preparation, the results confirm the potential of ZFAC as a purifier for dye removal from polluted water.

## 1. Introduction

Over the last few years, water dirtiness has become a global concern, being a major threat to human health and the environment. Water pollutants can be chemicals, trash, and microbes. Among the many chemicals present in polluted water are organic substances, of which dyes are considered the worst and certainly necessitate urgent solutions [1]. Water cleanliness is interrupted by the pollutants emerging out of several human activities. The rise in urbanization, industrialization, and improper discharge of wastes were found to be the major reasons for aquatic pollution [2,3]. Hence, manufacturing of papers, textiles, leathers, foods, plastics, pharmaceuticals, cosmetics, and printings generally are associated with effluents of large quantities of dyes due to their utilization in products’ colorization. Color is considerably the utmost obvious indicator of water pollution, being visible in the environment even at low dye quantities. Such pollution, caused by colorants, is a result of dyes’ high water solubility together with low degradability under natural conditions [4]. In aquatic environments, color reduces light penetration and further affects the photosynthesis of aquatic organisms.

**Figure 1:**
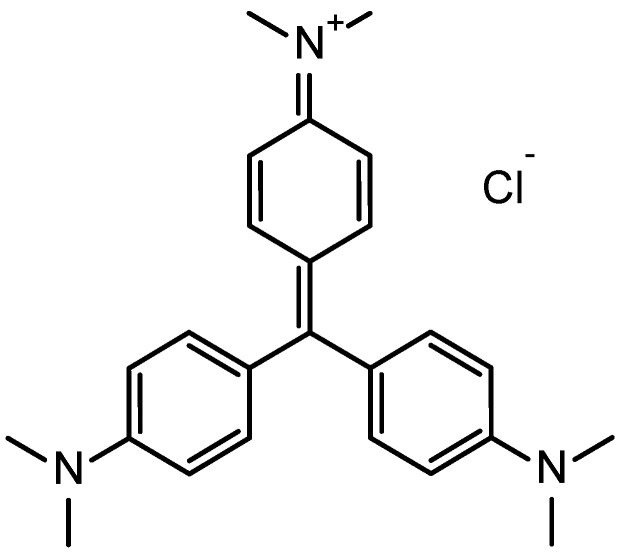
Chemical structure of cationic dye crystal violet (CV).

Crystal violet (CV) or gentian violet (Figure 1) is a basic dye that typically forms cationic salt upon dissolution in water [5]. It is known that cationic dyes are more toxic than anionic ones, perhaps because of their ability to interact with negatively charged cell membranes [6]. Among the many applications of CV, its usage for coloring things, biological staining, and veterinary and medical purposes is popular. However, it can cause skin and digestive tract irritation and, in extreme cases, it may lead to kidney failure and permanent blindness and, further, may exhibit carcinogenic effects [7,8,9,10,11]. Therefore, the removal of dyestuff from aquatic environments has become of special concern to specialists.

Several techniques have been utilized for remediation of dyes from polluted water such as adsorption-based strategies, membrane filtration, photodegradation, coagulation, chemical oxidation, and biological processes [12,13,14,15]. While each technique achieves varying degrees of success, it also has its own set of limitations. The adsorption method, which is simple, cost-competitive, and easy to operate, is the preferred, most popular, and affordable to developing nations [16]. Among the many introduced adsorbents, carbon-based materials are conventional and effective [17]. Nevertheless, factors such as the cost, capacity, and reusability may limit and value material suitability for application. A special interest has been given to activated carbons (AC), not only because of their structural properties but also due to their production simplicity, high adsorption capacity, selectivity, and fast processing [18,19]. Even though AC efficiency in dye remediation is feasible, it may be worth advancing its properties further [20].

In this present study, activated carbon was modified with ZnFe_2_O_4_ by simple impregnation method. The obtained AC/ZnFe_2_O_4_ (termed ZFAC) nanocomposite was fully characterized and explored as a potential adsorbent for the removal of cationic CV dye from aqueous solutions. The operating variables such as the pH of polluted solution, pollutant concentration, system temperature, adsorbent dosage, and contact time were investigated in batch method. Based on the kinetic, isothermal, and thermodynamic parameters, ZFAC’s adsorbent performance and adsorption mechanism were discussed.

## 2. Materials and Methods

### 2.1. Materials

Activated carbon (AC, decolorizing powder), zinc acetate dihydrate (Zn(CH_3_COO)_2_·2H_2_O) (98.9%), and crystal violet (CV, >90%), which was dried at 80 °C for about 2 h before use, were purchased from BDH Chemicals Ltd. (Poole, England, UK). Ferric nitrate nonahydrate (Fe(NO_3_)_3_·9H_2_O) (99.99%) and polyvinylpyrrolidone (PVP. (C_6_H_9_NO)x) powder were obtained from Sigma-Aldrich (St. Louis, MO, USA). Ethanol (EtOH, 99.5%), hydrochloric acid (HCl, 36%), and sodium hydroxide (NaOH, 98%) were bought from Fisher Chemical (Loughborough, UK). Unless otherwise stated, materials were used as received, and deionized water was used wherever necessary.

### 2.2. Synthesis of ZnFe_2_O_4_

ZnFe_2_O_4_ nanoparticles were synthesized in the presence of polyvinylpyrrolidone (PVP) as an organic spacer. Typically, 2 g of PVP was dissolved in 50 mL deionized water at 50 °C. After complete dissolution, the solution was brought to room temperature and a solution of Fe(NO_3_)_3_·9H_2_O (4.04 g, 0.01 mol) and Zn(CH_3_COO)_2_·2H_2_O (1.09 g, 0.005 mol) in 10 mL deionized water was slowly added with stirring, then left to stir for an additional 60 min at room temperature. Subsequently, the mixture was poured into a Teflon plate and dried at 60 °C for 24 h. Next, the dried metal oxide–polymer composite was hand ground to fine powder using mortar and pestle tools followed by calcination at 500 °C for 5 h under air atmosphere.

### 2.3. Preparation of AC/ZnFe_2_O_4_ (ZFAC) Nanocomposite

The metal oxide-activated carbon adsorbent, ZFAC nanocomposite, was prepared using a wet homogenization method in deionized water. Thus, ZnFe_2_O_4_ (15 wt% of the composite) was mixed with activated carbon (AC) in deionized water (30×, by weight, with respect to composite) with vigorous stirring for 15 min, followed by sonication for 3 h. The obtained composite (termed as ZFAC) was filtered, washed three times with water, and dried at 80 °C for 24 h. It is important to emphasize that various ratios of ZnFe_2_O_4_-to-AC, ranging between 5 and 30 wt%, were prepared, and their efficiency in the removal of cationic (methylene blue (MB) and crystal violet (CV)) and anionic (methyl orange (MO)) dyes was examined in the preliminary experiments; however, the ratio 15:85 was satisfactory, being more effective against cationic substances than anionic ones. Accordingly, following experiments were designed to investigate the efficiency of ZFAC (15:85 wt%) in the removal of CV dye. For comparison, the adsorption performance of individual metal oxide and AC in the removal of CV was also tested in the pilot test.

### 2.4. Characterization

The FTIR spectra were recorded on a Nicolet iS10 spectrophotometer (Thermo scientific, Madison, WI, USA) using the KBr–disc technique. Spectra were taken on the wavenumber range of 400–4000 cm^−1^ with 4 cm^−1^ wavenumber resolution and 32 runs per spectrum. X-ray diffraction (XRD) was measured in a Rigaku XtaLAB mini II benchtop X-ray crystallography system (The Woodlands, TX, USA) with copper Kα radiation (λ = 1.5418 Å) and a scan speed of 3°/min over two-theta (2θ) range of 10–90. Field emission scanning electron microscope (FESEM) micrographs were captured on a JSM-7610F LV SEM (JEOL, Tokyo, Japan) at an accelerating voltage of 15 kV. Thermogravimetric (TGA) analysis was performed using a Mettler Toledo TGA/DSC 1 Star system (Columbus, OH, USA). Samples (5–10 mg) were heated from 25–800 °C at a heating rate of 10 °C/min under nitrogen flow of 20 mL/min. Curves of thermal decomposition were depicted for TGA weight loss percentage and derivative-TGA (DTG). Surface area measurements were obtained from nitrogen adsorption isotherms at 77 K using NOVA 2200e surface area analyzer (Quantachrome Corp., Boynton Beach, FL, USA). Prior to measurement, samples were vacuum degassed at 180 °C for at least 2 h. The BET (Brunauer–Emmett–Teller)-specific surface area was calculated at P/P_0_ = 0.05–0.25, and the pore size distribution and volume were derived from the adsorption branch by using the Barrett–Joyner–Halenda (BJH) model. The electronic spectra were acquired using a double-beam UV–Vis spectrophotometer (U-2910, Hitachi, Tokyo, Japan) on the range of 200–600 nm, while the dye concentration was measured at λ_max_ of 590 nm at room temperature (23 ± 1 °C). The calibration curve was established using standard concentrations of CV on the range of 1–10 mg/L (*R*^2^ = 0.9914).

The pH at which the surface net charge of the prepared ZFAC adsorbent is zero (pH_PZC_) was studied using the pH drift method [21]. Hence, 10 mg of the adsorbent was added to 15 mL of 0.1 M NaNO_3_ solution of various initial pHs (pH_i_) ranging from 3 to 11. The pHs of the solutions were adjusted using 0.1 M of either HCl or NaOH, which were monitored using a bench-top pH-meter (Orion 3-Star from Thermo Scientific (Beverly, MA, USA) previously calibrated for the test range) before addition of the adsorbents. The mixtures were shacked at 100 rpm at room temperature (23 ± 1 °C) overnight, then centrifuged, and the final pHs (pH_f_) of the solutions were measured. The pH_PZC_ was assessed by plotting the change in the pH values before and after addition of the adsorbent (ΔpH = pH_f_−pH_i_) versus the pH_i_, and the point crossing the zero ΔpH was considered the pH_PZC_. The reported values are the average of two independent experiments.

### 2.5. Adsorption Experiments

The target adsorbent was affirmed after pre-experiment adsorption efficacy screening performed on various ZnFe_2_O_4_-loaded AC nanohybrid composites (0, 10, 15, 20, 25, and 30 wt% of ZnFe_2_O_4_) using 30 mg adsorbent dose, 100 ppm adsorbate concentration, 150 rpm agitation speed, unadjusted pH of the dye solution in distilled water, room temperature, and contact time of 24 h. Then, the best performing adsorbent (15 wt% ZnFe_2_O_4_-loaded AC, termed ZFAC) was further tested for its performance on the removal of cationic (CV and MB) and anionic (MO) dyes and compared with the unloaded AC under the screening conditions. Accordingly, the experiments herein were decided to be accomplished further.

The adsorption experiments were carried out, unless otherwise stated, under the following conditions: adsorbate (CV) initial concentration 100 ppm, adsorbate volume 50 mL, adsorbent (ZFAC) dose 30 mg, agitation speed 150 rpm, room temperature 23 ± 1 °C, solution pH 7.2, and adsorption duration of 24 h. Parameters affecting the adsorption efficiency were optimized in the following order. First, the pH effect was investigated on the range from 3 to 11, adjusted to the target values through dropwise addition of 0.1 M HCl or NaOH, and monitored with the help of pH-meter. Second, the optimal adsorption dosage was obtained by screening the adsorption efficiency on the range 10–50 mg, keeping other parameters mentioned above fixed. Third, the influence of adsorbate initial concentration was studied over a range of 25–200 mg/L. Finally, the effect of temperature and adsorption thermodynamic parameters was determined by applying adsorption at room temperature (23 ± 1 °C), 35, and 45 °C. Focusing on the kinetic behavior, the adsorption kinetics were investigated at selected expressive initial concentrations of 25, 50, and 100 ppm using the optimized condition.

### 2.6. Desorption Experiments

The reusability of ZFAC adsorbent was also tested. The adsorption was performed for a 100 ppm CV concentration at the optimal condition. The adsorbent was collected by filtration, fairly washed with distilled water, dried at about 40 °C overnight, weighed, and used for desorption process. Regeneration experiment was carried out in batches of 5 mL × 5 time, using ethanol–water solution (25%) [22]. After desorption, the adsorbent was dried as above until constant weight, then used for the next cycle. The process was repeated for three cycles of sorption–desorption processes.

### 2.7. Adsorption Modeling

A stock solution of CV at a concentration of 500 ppm was prepared in deionized water from which the working solutions were obtained by dilution. The dye concentration to be analyzed using the UV–Vis method was determined in reference to an established standard curve. According to the Beer–Lambert law, the extension coefficient at 590 nm (λ_max_) was found to be 0.1436 ppm^−1^ cm^−1^ (58,575 M^−1^ cm^−1^) at room temperature.

#### 2.7.1. Adsorption Isotherms

The removal efficiency (*Re*, %) and adsorption capacity (mg/g) at time *t* (*q_t_*) and at equilibrium (*q_e_*) were calculated according to Equations (1)–(3), respectively.

(1) $$Re\%=\left(\frac{C_{0}-C_{e}}{C_{0}}\right)100$$

(2) $$q_{t}=\frac{\left(C_{0}-C_{t}\right)V}{m}$$

(3) $$q_{e}=\frac{\left(C_{0}-C_{e}\right)V}{m}$$

where *C*_0_, *C_t_*, and *C_e_* (mg/L) are the adsorbate initial concentrations in the liquid phase and the ones at time *t* (min) and at equilibrium. *V* (L) is the solution volume, and *m* (g) is the dry mass of adsorbent used.

The adsorption isotherm was computed using the Langmuir [23,24], Freundlich [25], and Temkin [26] models, as given in Equations (4)–(6), respectively.

(4) $$q_{e}=\frac{Q_{m}K_{L}C_{e}}{1+K_{L}C_{e}}$$

(5) $$q_{e}=K_{F}C_{e}^{\frac{1}{n}}$$

(6) $$q_{e}=\frac{RT}{K_{T}}lnA+\frac{RT}{K_{T}}lnC_{e}=BlnA+BlnC_{e}$$

where *C_e_* and *q_e_* have the same meaning as above, *Q_m_* (mg/g) is the maximum monolayer capacity, and *K_L_* (L/mg), *K_F_* (mg/g)(L/mg)^1/n^, and *K_T_* (J/mol) are, respectively, the model isotherm constants; *T* (K) is the absolute temperature, *R* (8.314 J/mol·K) is the universal gas constant, and *A* (L/mg) is the Temkin isotherm equilibrium binding constant.

#### 2.7.2. Adsorption Thermodynamic

The thermodynamic parameters, including Gibbs free energy change (∆*G*°), enthalpy change (∆*H*°), and entropy change (∆*S*°), were analyzed following expressions given in Equations (7) and (8) [4,27].

(7) $$lnK_{d}=\frac{\Delta S{}^{\circ}}{R}-\frac{\Delta H{}^{\circ}}{RT}=-\frac{\Delta G{}^{\circ}}{RT}$$

(8) $$K_{d}=\frac{q_{e}}{C_{e}}$$

where *K_d_* (L/g) is the apparent equilibrium constant, while other notations have the same meaning as above.

#### 2.7.3. Adsorption Kinetics

The time-dependent adsorption study was performed at different initial dye concentrations (25, 50, and 100 ppm). The obtained data were fitted to the linear pseudo-first-order (PFO), pseudo-second-order (PSO), and intra-particle diffusion (IPD) models, described by Lagergren–Svenska [28], Ho–McKay [29], and Weber–Morris [30], respectively, as given by Equations (9)–(11).

(9) $$\log\left(q_{e}-q_{t}\right)=log(q_{e})-\frac{k_{1}t}{2.303}$$

(10) $$\frac{t}{q_{t}}=\frac{1}{k_{2}q_{e}^{2}}+\frac{t}{q_{e}}$$

(11) $$q_{t}=k_{id}t^{0.5}+C$$

where *k*_1_ (min^−1^), *k*_2_ (g/(mg·min)), and *k_id_* (mg/(g·min^0.5^)) are the rate constants of the three models, respectively, and *C* (mg/g) is the y-intercept of the IPD model. The reciprocal value of the PSO plot intercept describes the initial rate (*h*) (mg/(g·min)) of the adsorption process, as in Equation (12).

(12) $$h=k_{2}q_{e}^{2}$$

## 3. Results

### 3.1. Structural Characterization

#### 3.1.1. FTIR Analysis

**Figure 2:**
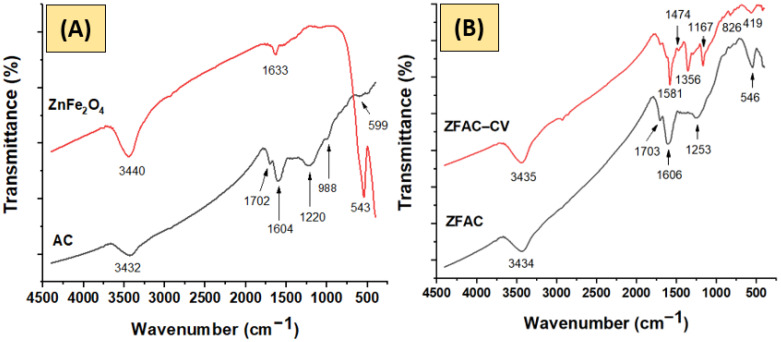
FTIR spectra of: (**A**) activated carbon (AC) and metal oxide (ZnFe_2_O_4_); (**B**) adsorbent (ZFAC) and crystal violet (CV)-loaded adsorbent (ZFAC–CV).

Figure 2 shows the FTIR spectra of AC, ZnFe_2_O_4_, ZFAC, and ZFAC–CV. As can be seen, the spectra share common bands for OH stretching and bending vibrations at about 3435 and 1605 cm^−1^, respectively. The latter, however, is broad due to overlapping, with major contribution from C=C stretching [31,32]. The AC spectrum displayed bands at 1702, 1604, 1220–1166, 988, and 599 cm^−1^ corresponding to νC=O stretching, νC=C stretching, νC–OH, asymmetric and symmetric νC–O–C, and structure deformation modes, respectively. The ZnFe_2_O_4_ spectrum revealed a dominant band at 543 cm^−1^, characteristic for Fe–O. The M-O bands can be traced in ZFAC and ZFAC–CV as well, thus indicating composite formation with a slight shift in the peak positions due to interactions. Although the Zn-O band is typically bellow 400 cm^−1^, which is, unfortunately, out of the recorded range, the traced peaks around 418 cm^−1^ in the spectra of ZFAC and ZFAC–CV could be assigned to Zn–O [33,34]. After adsorption (ZFAC–CV), additional peaks appeared, e.g., at 2929, 2862, 1474, and 1356 cm^−1^, attributed to the asymmetric and symmetric stretching of methyl group, N–H bending, and C–N stretching vibrations in the dye, respectively [35]. It could be seen that the broad band centered at 1605 in ZFAC was diminished after adsorption and a new, sharp, and red-shifted peak at 1581 cm^−1^ was observed. This suggests that C=O and OH are involved in the adsorption process, providing active sites for CV settlement.

#### 3.1.2. XRD Analysis

**Figure 3:**
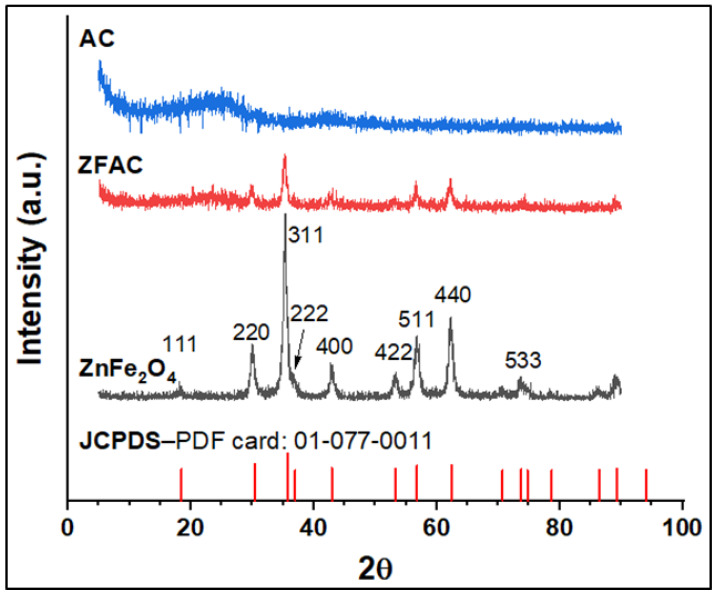
XRD diffractograms of ZnFe_2_O_4_ (JCPDS card), ZnFe_2_O_4_ (experimental), ZFAC, and AC.

Figure 3 presents the XRD patterns of the studied samples along with the JCPDS card of the metal oxide ZnFe_2_O_4_ for comparison. The diffractogram of the obtained spinel metal oxide NPs typically agrees with the literature [36,37] and JCPDS PDF (Card no: 01-077-0011), showing intense peaks at 2θ values of 18.44, 30.44, 35.74, 42.95, 53.28, 56.80, 62.36, and 73.64 corresponding to the planes (111), (220) (311), (422), (400), (511), and (440), respectively. Moreover, no other phases for impurity were found, confirming the structural integrity of the obtained ZnFe_2_O_4_ NPs. The resulting cubic ZnFe_2_O_4_ has a unit cell parameter of 8.423 Å, which is close to the reported values (8.438 and 8.389 Å) in the literature [38]. It is noteworthy that the broadening peak in the formed spinel structure is an indicative of nano-sized particles. The diffractogram of AC revealed two prominent broad peaks at about 2θ = 19–29° and 40–45° (centered around 2θ = 23.64 and 42.24 and corresponds to the plane 002 and 101), attributed to the amorphous carbon and α-axis of the graphitic structure, respectively [39,40]. The XRD profile of the ZFAC composite was also recorded, and the peaks corresponding to ZnFe_2_O_4_ were still identifiable; however, peak intensities were slightly diminished due to AC. All diffraction peaks were indexed to the cubic structure of spinel-doped metal oxide.

#### 3.1.3. BET Analysis

**Figure 4:**
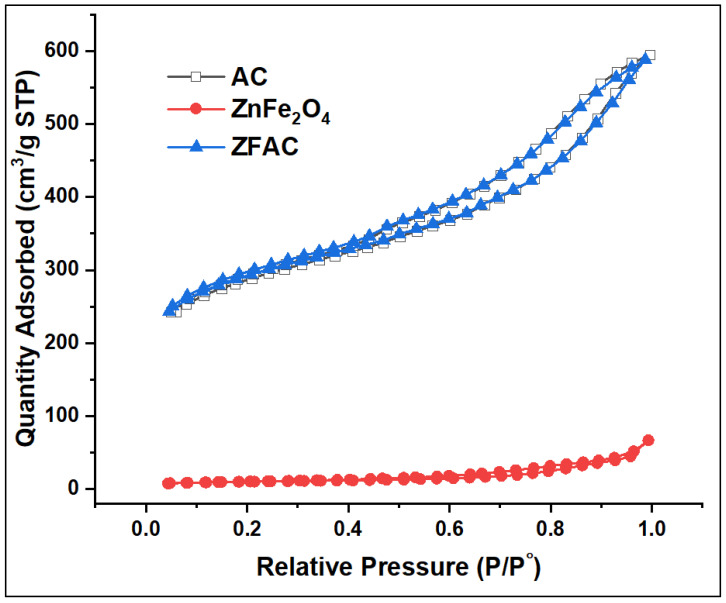
Nitrogen adsorption–desorption isotherms of activated carbon (AC), ZnFe_2_O_4_, and ZnFe_2_O_4_–AC (ZFAC).

Figure 4 shows the N_2_ adsorption–desorption isotherms of AC, ZnFe_2_O_4_, and ZFAC, while the corresponding surface area, pore size, and pore volume are summarized in Table 1. The BET surface area of ZFAC (948 m^2^·g^−1^) was close to that of AC (955 m^2^·g^−1^), while the one of ZnFe_2_O_4_ was as low as 34 m^2^·g^−1^, assuming a negligible effect of ZnFe_2_O_4_ on the overall surface area of the adsorbent. On the other hand, ZnFe_2_O_4_ has the highest BJH pore size and the lowest pore volume compared with both AC and ZFAC (Table 1). The instant high N_2_ adsorption at low relative pressures onto AC and ZFAC compared with ZnFe_2_O_4_ indicates their high uptake affinity, which is associated with their high content of micropores, and thus their high surface area [41].

#### 3.1.4. SEM Analysis

**Figure 5:**
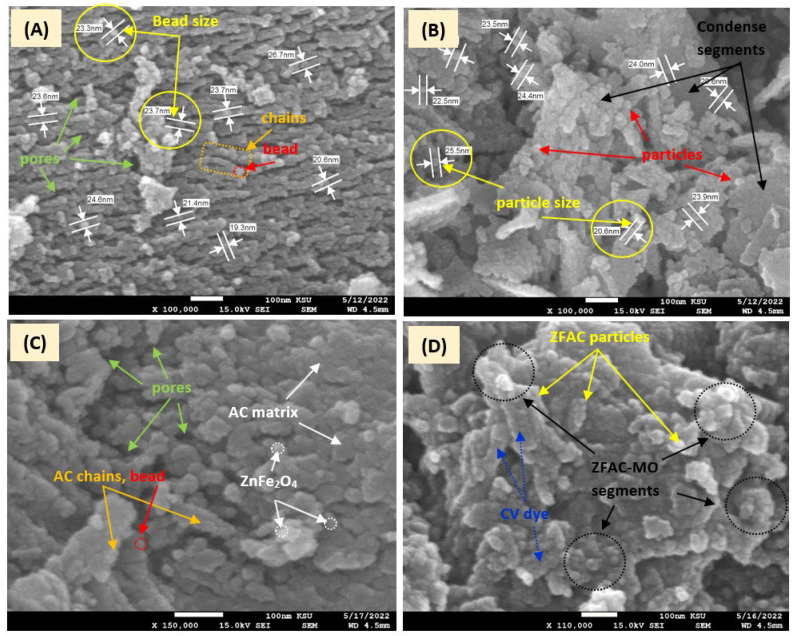
Scanning electron micrographs (SEM) of (**A**) activated carbon (AC); (**B**) ZnFe_2_O_4_; (**C**) ZFAC, free adsorbent; (**D**) ZFAC-CV, dye-loaded adsorbent. Scale bar 100 nm; magnification 100,000–150,000×; acceleration 15.0 kV.

The SEM micrographs of AC, ZnFe_2_O_4_, ZFAC, and ZFAC-CV are given in Figure 5. It can be seen that the AC particles are relatively ordered in chaplet-like chains with a bead average particle size of 22.99 nm and about 5–10 beads per chain. However, observed voids in between filaments regenerate considerable pores and may explain their high surface area. On the other hand, the surface morphology of the prepared spinel metal oxide (ZnFe_2_O_4_) is more condensed, with an average particle size of 23.59 nm, and arranged in more randomly adhered segments. After composition, the SEM image of the obtained ZFAC revealed a slight alteration of the AC surface structure, with less aggregations and shortened bead-like based chains. The SEM graph of CV-loaded adsorbent (ZFAC–CV), showed more adhesives particles, due to CV filling ZFAC cavities.

#### 3.1.5. TEM Analysis

**Figure 6:**
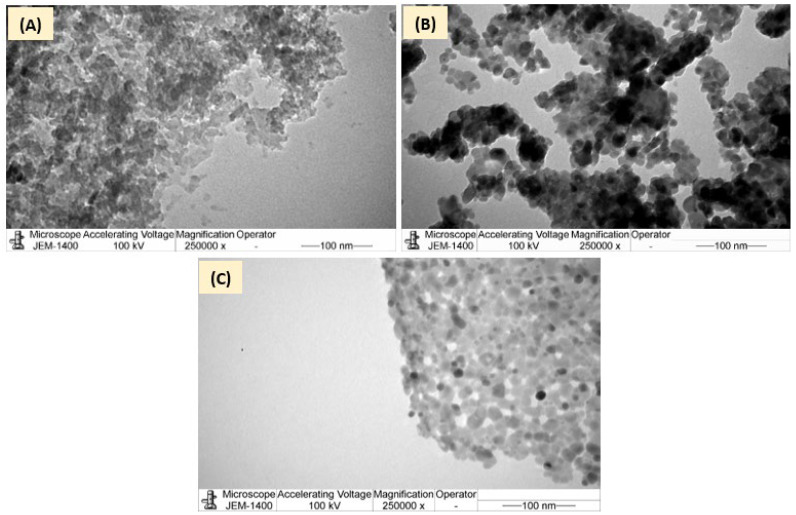
Transmission electron microscope (TEM) of (**A**) AC, (**B**) ZnFe_2_O_4_, and (**C**) ZFAC.

The TEM micrographs of AC, ZnFe_2_O_4_, and ZFAC were also recorded (Figure 6). The AC image revealed a disordered porous structure with large quantities of white spots between the disordered layers, which suggest the existence of micropores and mesopores that provide enough space for CV to settle [42]. In Figure 6B, the TEM morphology of ZnFe_2_O_4_ revealed moderate aggregation of primarily particles with counted particle sizes (*n* = 22) of 14.30 ± 4.34 nm, which is less than that suggested by SEM. Such structural morphology of ZnFe_2_O_4_ was also seen by Abbasian et al. [36], and the particle sizes are close to that reported by Wang and Shih (12.1 nm) [43]. Figure 6C shows TEM image of ZFAC nanocomposite in which a relatively uniform distribution of metal oxide nanoparticles on the AC surface can be observed. It seems that the composition led to less aggregates for both AC and ZnFe_2_O_4_ nanoparticles, providing comparative surface area with higher active sites, supporting the observed sorption enhancement of ZFAC nanocomposite compared with AC.

The low BET surface area of ZnFe_2_O_4_ (34 m^2^·g^−1^) compared with AC (955 m^2^·g^−1^) is a result of its particles’ high agglomeration, which led to less porosity per g of ZnFe_2_O_4_, as can be seen in the SEM and TEM micrographs as well.

#### 3.1.6. TGA Analysis

**Figure 7:**
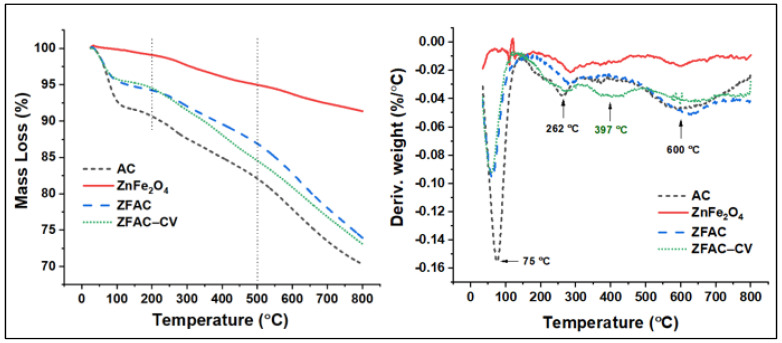
Thermogravimetric (TGA) thermograms (left) and their corresponding derivative (DTG) curves of AC, ZnFe_2_O_4_, ZFAC, and ZFAC–CV.

The thermal property of AC and ZnFe_2_O_4_ alone, the adsorbent ZFAC nanocomposite, and the dye-loaded ZFAC were further examined. As shown in Figure 7, the thermograms can be divided into three regions, 25–200–500–800 °C. The first region is mainly a result of the desorption of weakly adsorbed water and gases. The second (200–500 °C) is due to decomposition of organic matters either, e.g., matrix residues in ZnFe_2_O_4_ or organic-based backbone in ZFAC and ZFAC–CV. The last region (typically above 600 °C) is due to the decomposition of functional groups and partial gasification of the least thermally stable fragments of the carbon structure [44]. An AC thermogram revealed a major mass loss in the first step (10 wt%), then 8 wt% in the second, leaving a mass residue of 70% at 800 °C. The TGA curve of ZnFe_2_O_4_ shows a relatively high thermal stability; however, the observed total mass loss of about 8.5 wt% at 800 °C could be ascribed to organic matrix (PVP) residues, a spacer used to facilitate production of ZnFe_2_O_4_ NPs [45]. The curves corresponding to the adsorbent before and after adsorption have similar patterns for the first step of decomposition (5.5 wt% weight loss), while slightly differ afterword. At the end of stage 2, the total mass loss from both ZFAC and ZFAC–CV were found to be 7.7 and 10 wt%, with the difference (2.3 wt%) attributed to the adsorbed CV mass. The calculated residual masses at 800 °C were, respectively, 73.9 and 73.1 wt% (Δ = 0.8 wt%). Meanwhile, the TGA thermograms present the mass loss under dynamic temperature; DTG curves can also help in the presentation of the mid-point of transition/decomposition. As shown in Figure 7, the three stages of TGA decomposition showed DTG peaks around 70, 260, and 630 °C. However, an additional degradation peak in ZFAC–CV was observed approximately at 400 °C and assigned to CV fraction. This agreed with the literature [46], in which the CV molecule decomposed above 190 °C with an off-set value around 520 °C; the observed difference in the values of the on-set and off-set of decomposition may be a result of components’ interaction and the existence of various organic matters that decompose simultaneously.

### 3.2. Batch Adsorption Study

#### 3.2.1. Pre-Evaluation of Prepared Adsorbents

**Figure 8:**
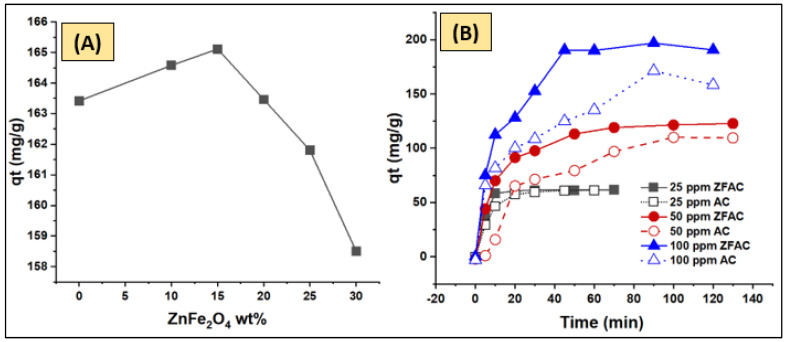
(**A**) Adsorption performance of various ZnFe_2_O_4_/activated carbon (ZFAC) nanocomposites. (**B**) Time profile of crystal violet (CV) (25, 50, and 100 ppm) adsorption onto AC and ZFAC adsorbents.

The adsorption performance of various composites was also analyzed. ZFAC composites containing 0, 10, 15, 20, 25, and 30 wt% of ZnFe_2_O_4_ were prepared and applied for removal of CV dye from water solutions. As shown in Figure 8A, the adsorption capacity was optimal for the ZFAC containing 15 wt% ZnFe_2_O_4_, reflecting the rule, as well as the benefit, of ZnFe_2_O_4_ in decorating the AC surface for CV settling [31]. This implies that some chemical/physical and, possibly, mechanical interactions have led to surface modification at low ZnFe_2_O_4_ wt% and up to 15 wt%, beyond which the adsorptive properties of ZnFe_2_O_4_ dominate as a result of quantity increase. It is worth mentioning that ZnFe_2_O_4_ alone has low activity as an adsorbent for CV dye removal as examined during the pilot studies. Moreover, it is reported that ZnFe_2_O_4_ with certain structural morphologies and particle sizes may have photocatalytic properties [47,48]. Thus, to ensure that no catalytic CV degradation by ZnFe_2_O_4_ occurs, batch experiments of pure AC, pure ZnFe_2_O_4_, and ZFAC nanocomposite were separately performed under the adsorption operating condition (30 mg adsorbent, 50 mL of 100 ppm adsorbate, 2 and 24 h agitation time, 150 rpm agitation speed, room temperature, and normal laboratory lighting that automatically shut down overnight). As stated above, there was no change in the CV concentration for the system containing ZnFe_2_O_4_ alone, indicating negligible adsorption and further confirming no catalytic effect at the adsorption condition. The time-dependent adsorption of CV onto AC and ZFAC is illustrated in Figure 8B. It is clear that the capacity of ZFAC was higher than that of AC, reaching equilibrium faster. Such results further support the benefit of metal oxide in the modification of the AC surface toward enhancement of its adsorptive properties of cationic dyes including CV.

#### 3.2.2. Effect of pH_PZC_ and pH

**Figure 9:**
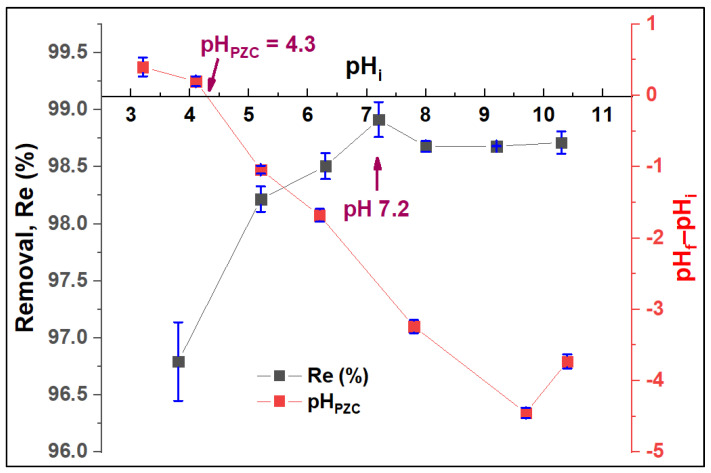
Effect of pH on the adsorption process of CV by ZFAC and the pH at surface zero charge (pH_PZC_) of ZFAC adsorbent. Bars represent the standard error of the mean (*n* = 2).

The adsorbent surface charge was assessed using the pH-drift method. The pH_PZC_ value was found at 4.3 (Figure 9). pH_PZC_ typically determines the combined influence of all the functional groups of the ZFAC surface. At pH < pH_PZC_, the surface charge is positive, while at pH > pH_PZC,_ the surface has a net negative charge. Figure 9 also shows the adsorption efficiency at different pH values and indicates a pH-dependent process. Hence, the removal has increased with pH increase, reaching the maximum value at pH 7.2, then slightly decreasing and becoming stable above pH 8. At low pH, the H^+^ ions in the adsorption solution are abundant and thus compete with CV molecules on the adsorbent active sites [49], leading to a substantial increase in the efficiency up to the pH_PZC_ value (pH 4.3). With pH increase from 4.3 to 7.2, the available negatively charged sites for CV residency become plentiful, reaching the maximum at pH of 7.2. Next, the adsorption was slightly decreased with a pH increase from 7.2 to 8, above which (up to pH 10.5,) the pH has no visible effect on the *Re*%. However, the removal was sufficient all over the investigated pHs, with *Re*% ranging between 99.0 and 96.5%.

#### 3.2.3. Effect of Adsorbent Dosage

**Figure 10:**
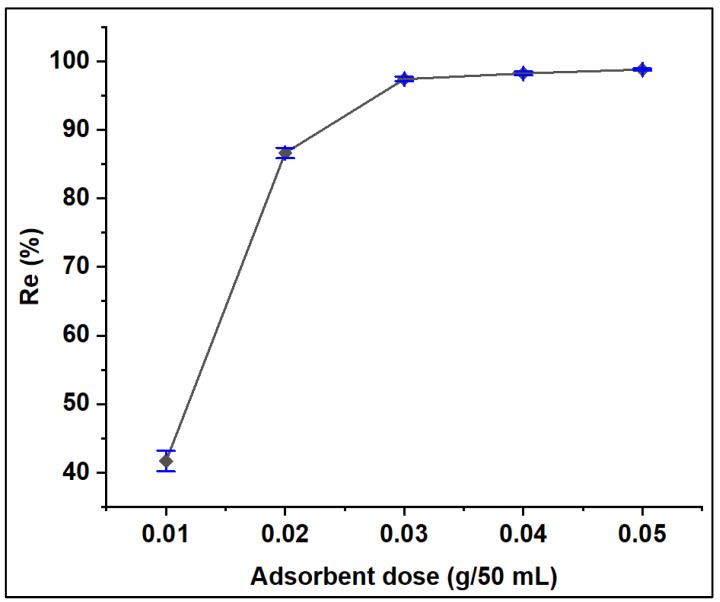
Effect of adsorbent dose on the adsorption of CV onto ZFAC. Bars represent the standard error of the mean (*n* = 2).

The performance of various ZFAC doses on the removal of CV dye from its aqueous solution was examined for a series of 0.2–1.0 g/L (10–50 mg adsorbent per 50 mL dye solution) keeping other parameters constant (Section 2.4). The results, expressed in terms of adsorption efficiency (*Re*, %), are illustrated in Figure 10. It can be seen that the *Re*% increased as adsorbent doses increased from 0.01 to 0.03 g, then, no notable difference in removal efficiency can be seen up to 0.05 g. The general enhancement could be attributed to the increase in the number of available active sites reaching maxima at 0.03 g, beyond which the available sites for adsorption decrease due to overlapping or aggregation [50].

#### 3.2.4. Adsorption Isotherms

**Figure 11:**
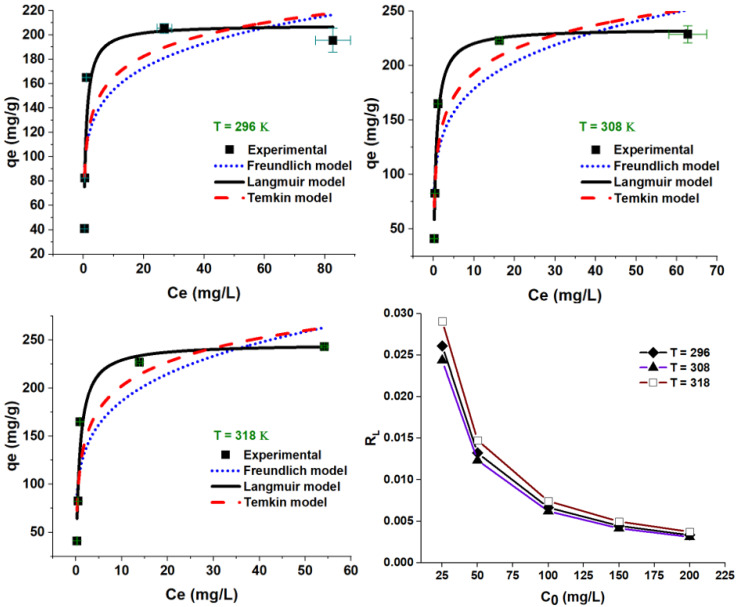
Langmuir, Freundlich, and Temkin plots for CV adsorption onto ZFAC. Bars represent the standard error of the mean (*n* = 2).

The adsorption equilibrium was evaluated at temperatures of 23, 35, and 45 °C and modeled using the Langmuir, Freundlich, and Temkin isotherms (Equations (4)–(6)). As shown in Figure 11 and Table 2, the adsorption capacity increased with temperature increase, suggesting a temperature-dependent process. According to the values of the coefficient of determination, *R*^2^, (e.g., at 296 K), the goodness of fit for the experimental data was found to be in the order of Langmuir (*R*^2^ = 0.864), Temkin (*R*^2^ = 0.724), and Freundlich (*R*^2^ = 0.663); the same trend was kept at 308 and 318 K. The maximum theoretical adsorption capacities (Qm, mg/g) were greater than the experimental ones, increased from 208.29 to 246.19 mg/g as temperature increased from 296 to 318 K, thus demonstrating favored adsorption at higher temperature. The Langmuir dimensionless constant R_L_ was also calculated (Equation (13)), drawn in Figure 11, and tabulated in Table 2 as well. Such a factor is one indication of adsorption favorability: *R_L_* = 0 (irreversible), 0 < *R_L_* < 1 (favorable), *R_L_* = 1 (linear), and *R_L_* > 1 (unfavorable). As seen in Table 2, the *R_L_* values support favorable adsorption all over the investigated ranges of concentrations and temperatures. Moreover, the tendency of adsorption equilibrium can also be assessed using 1/*n*; the Freundlich heterogeneity factor with 0.1 < 1/*n* < 1.0 values support a favored sorption process.

(13) $$R_{L}=\frac{1}{1+K_{L}C_{0}}$$

#### 3.2.5. Adsorption Thermodynamics

**Figure 12:**
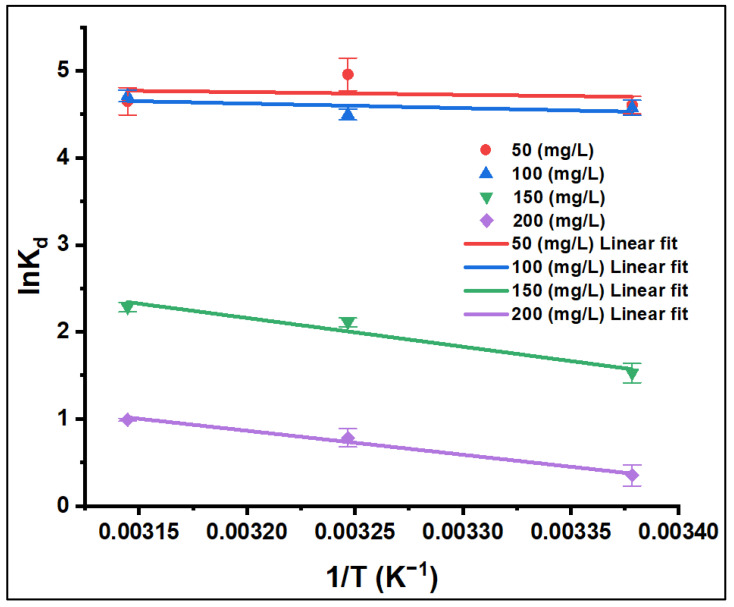
Effect of temperature on the adsorption of CV onto ZFAC. Bars represent the standard error of the mean (*n* = 2).

The effect of temperature on the adsorption process of CV onto ZFAC was studied in batch at three temperatures (23, 35, and 45 °C) and for four initial CV concentrations (*C*_0_ = 50, 100, 150, and 200 mg/L). The thermodynamic behavior was evaluated using Equation (7), and the results are depicted in Figure 12 and summarized in Table 3. In all cases, the sign of Δ*G*° was negative, directing the feasibility and spontaneity of the sorption process. As temperature increased from 23 to 45 °C, the extent of Δ*G*° negativity was slightly increased, whereas it decreased as CV concentration increased from 50 to 200 mg/L. As seen in Table 3, the positive values of Δ*H*° and Δ*S*° endorse the endothermic nature of the adsorption process, and randomness increases at the solid–liquid interface. Although Δ*S*° and Δ*H*° were virtually temperature-independent [51], the spontaneity was temperature-dependent, which further supports the mobility of adsorbate molecules, and thus their affinity for adsorption is higher at higher temperature [52,53].

The adsorption mechanism can be assessed through the magnitudes of the thermodynamic parameters. According to Table 3, the sorption process is favored at high temperatures and at low adsorbate concentrations. As the values of Δ*G*° and Δ*H*° were on the range of physical processes (i.e., Δ*G*° from −20 to 0 kJ/mol and Δ*H*° less than 40 kJ/mol), the mechanism for adsorption of CV onto ZFAC is predominantly physical in nature [4].

#### 3.2.6. Kinetic Study—Contact Time of Various CV Concentrations

**Figure 13:**
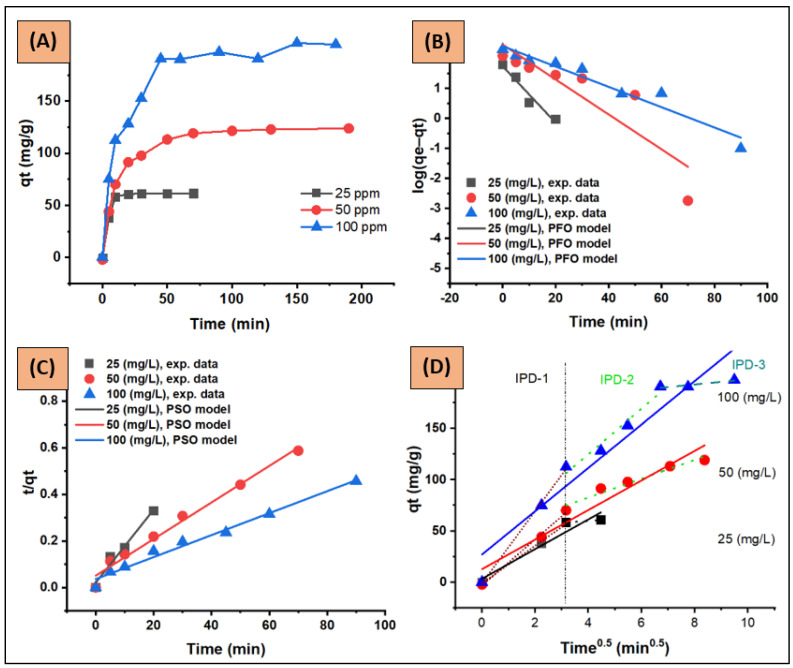
(**A**) Adsorption kinetic profile, (**B**) pseudo-first-order (PFO), (**C**) pseudo-second-order (PSO), and (**D**) intra-particle-diffusion kinetic plots (IPD, IPD stages were marked as IPD-1, IPD-2 and IPD-3) for CV adsorption onto ZFAC. Conditions: adsorbate intial concentration *C*_0_ = 25, 50, and 100 ppm; *C*_0_ volume = 250 mL; adsorbent dose = 0.1 g; tepmerature = 23 °C; pH = 7.2; shaking speed = 150 rpm.

Figure 13A illustrates the time profile of CV adsorption onto ZFAC adsorbent for initial concentrations of 25, 50, and 100 ppm, and the applied kinetic models (PFO, PSO, and IPD) are shown in Figure 13B–D. The values of the corresponding kinetic parameters, constants, and correlation coefficients are given in Table 4 and Table 5. As can be seen, the adsorption rate was fast at the initial phase, indicating the presence of an abundance of readily available active sites on the adsorbent surface. Next, the adsorptions continued at a slower rate until saturation, which was assigned beyond 10 min for 25 ppm and 70 min for 50 and 100 ppm. The adsorbent capacity (*q_e_*, mg/g) was found to be increased as initial dye concentration increased from 25 to 100 ppm (Figure 13A). According to *R*^2^ (Table 4 and Figure 13B,C), the PSO equation fitted the kinetic data better than PFO, with the experimental *q_e_* much closer to PSO as well. The constants *k*_1_ and *k*_2_ were inversely proportional to dye *C*_0_, demonstrating that the adsorbent surface saturation depends on *C*_0._ Hence, at low *C*_0_, dye molecules can interact with the adsorbent binding sites with less competition, resulting in higher rate constants; but, at higher *C*_0_, molecules may need to diffuse to the internal sites by intra-particle diffusion, resulting in reduced rates [50,54]. The *h* constant, which express the initial adsorption rate, can be obtained from the PSO equation when *t* approaches zero (Equation (12)). As seen in Table 4, the values of *h* were *C*_0_-dependent, being lower at higher *C*_0_. However, the increase in *C*_0_ led to an increase in the loading capacity of the adsorbent, possibly due to the higher driving force for mass at higher *C*_0_ [55].

Since neither PFO nor PSO models could assess the diffusion mechanism and rate-limiting step, the IPD model described by the Weber–Morris equation (Equation (11)) was applied as well. This model assumes that the adsorption can occur through three sequential steps: (1) film diffusion, in which the external adsorbate molecules transfer across the liquid film to the adsorbent exterior surface; (2) intra-particle diffusion, the transport of adsorbent particles from its surface to its internal pores; and (3) sorption, the interaction step. Step (3) is known to be fast and cannot be treated as a rate-limiting step, thus the rate-controlling step can be (1) and/or (2) [4,56]. According to the W–M plot (Figure 13D), the deviation of linearity from the origin (0, 0) suggests, in addition to intra-particle diffusion, the involvement of other mechanisms, including film diffusion, in the adsorption process. The obtained values of the intercept *C* (Table 5) were relatively high (3.534, 12.911, and 27.166 at 25, 50, and 100 ppm, respectively) and increased with *C*_0_ increase, indicating unignorable boundary film thicknesses. As the IPD plots were carved, the diffusion mechanism could be divided into various linear stages as illustrated in Figure 13D and Table 5. According to the literature [4,57], as the *k_id_* decreased from the first stage to the next (e.g., for *C*_0_ = 100 ppm, *k_id_*-S1, *k_id_*-S2, and *k_id_*-S3 were 35.13, 22.11, and 2.48, respectively) the uptake is mainly film-diffusion-controlled.

### 3.3. Reusability of ZFAC

**Figure 14:**
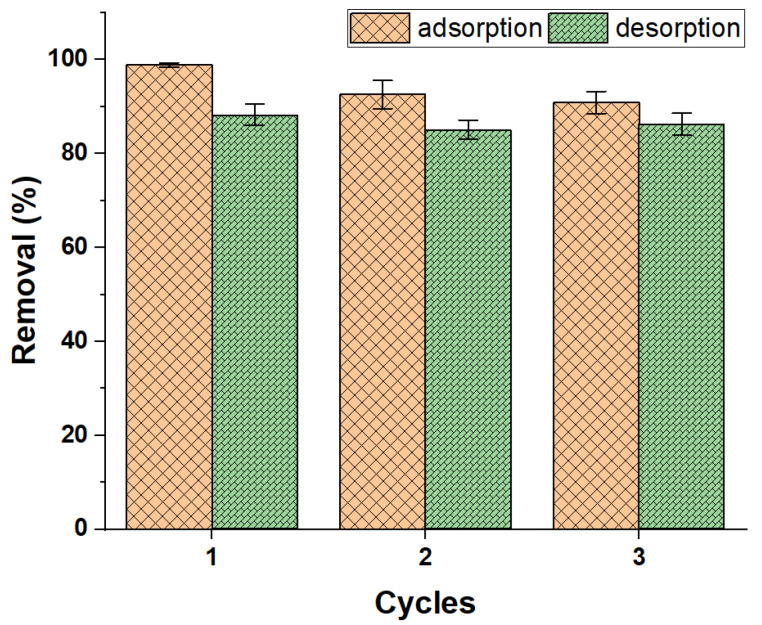
Adsorption efficiency of ZFAC after three cycles of operation. Bars represent the standard error of the mean (*n* = 2).

The reuse study tried to prove the recycling ability of the prepared ZFAC adsorbent. It was found that the dye might be desorbed from ZFAC nanoparticles with diluted ethanol up to 86.3% after three cycles of the adsorption–desorption process (Figure 14). However, the efficiency was slightly reduced after cycle 1, possibly due to surface alteration caused by adsorption operation, otherwise being affected by the eluent used, a case that may necessitate further optimization of eluents.

### 3.4. Relative Performance of ZFAC

The performance of ZFAC in CV dye removal compared with some other adsorbents taken from the literature is given in Table 6, using the maximum capacity (*Q_m_*, mg/g) as the base for comparison. The compared adsorbents represent a variety of materials close to the investigated ZFAC and selected as metal oxides/activated carbon composites [58,59,60,61], metal oxide [58], commercial activated carbon [62], bio-based activated carbons [50,63], and synthetic activated carbon [21]. For easy comparison, the adsorption conditions were also tabulated. As can be seen, the *Q_m_* value of ZFAC at the operating condition is 208.3 mg/g, which is relatively higher than the alike composites shown in Table 6. The reported *Q_m_* of nanomagnetic iron oxide is low (12.7 mg/g), which could support the observed negligible results in this work. On the other hand, synthetic activated carbons may be more efficient than commercial and bio-based ones, while ZFAC nanocomposite performance is still comparatively high. However, the differences in adsorption properties and adsorbent performance are a result of various factors, including adsorbent nature and properties such as morphology and particle size, adsorbate structure and ionization state, and adsorption operating conditions. The suitability of a potential adsorbent is generally assessed in terms of efficiency, reusability, and availability. Thus, both naturally and industrially based adsorbents may be inexpensive but commonly less efficient. By some engineered modification, adsorbent performance commonly improved, validating the advantage of metal oxides’ (i.e., ZnFe2O4) addition to activated carbon, as discussed for ZFAC.

## 4. Conclusions

In this work, an activated carbon/ZnFe_2_O_4_ (ZFAC) nanocomposite was successfully prepared by simple wet homogenization method. A composition containing 15 wt% ZnFe_2_O_4_ exhibited adsorption properties superior to its pure precursors, ZnFe_2_O_4_ and AC, in terms of remediation of cationic-dye-polluted water, CV dye in particular. The as-combined adsorbent nanoparticles were characterized by FTIR, XRD, SEM, N_2_ adsorption-based surface area, and TGA. Results of adsorption indicate a pH-dependent process with efficiency increased with temperature and dye concentration increase, while adsorbent dosage increase promotes efficiency reduction. The adsorption process followed Langmuir and pseudo-second-order equations with a maximum capacity of, e.g., 208.29 mg/g at 23 °C and a rate-limiting step controlled by a film-diffusion mechanism. The overall adsorption mechanism, assessed by the values of thermodynamic parameters including Δ*G*°, Δ*H*°, and Δ*S*°, was physical and endothermic, operating with randomness increase at the adsorption interface. This result supports the advantage of such a system in the enhancement of activated carbon adsorptive properties towards application for the removal of selected organic pollutants such as cationic CV dye.

## Figures and Tables

**Table 1:** BET surface area, pore size, and pore volume of AC, ZnFe_2_O_4_, and ZFAC.

Sample	BET Surface Area (m^2^·g^−1^)	Pore Size (nm)	Pore Volume (cm^3^·g^−1^)
AC	955	1.96	0.546
ZnFe_2_O_4_	34	4.26	0.097
ZFAC	948	1.97	0.524

Abbreviations: AC, activated carbon; BET, Brunauer–Emmett–Teller-specific surface area; ZFAC, ZnFe_2_O_4_–AC adsorbent.

**Table 2:** Langmuir, Freundlich, and Temkin isotherm parameters for CV adsorption onto ZFAC.

Temp. (K)	Langmuir				Freundlich				Temkin		
	*Q_m_* (mg/g)	*K_L_* (L/mg)	*R_L_* (C0 (mg/L) = 25, 50, 100, 150, 200)	*R* ^2^	*K_F_*	*n*	*1/n*	*R* ^2^	*K_T_*	*b*	*R* ^2^
296	208.29	1.49	0.026, 0.013, 0.007, 0.004, 0.003	0.864	107.46	6.30	0.159	0.663	74.35	42.77	0.724
308	234.03	1.60	0.024, 0.012, 0.006, 0.004, 0.003	0.980	116.62	5.40	0.185	0.798	44.29	35.07	0.882
318	246.19	1.34	0.029, 0.015, 0.007, 0.005, 0.004	0.945	116.94	4.93	0.203	0.795	28.98	32.17	0.869

Kinetic model terms: Langmuir: *Q_m_*, the maximum monolayer capacity of the adsorbent; *K_L_*, Langmuir constant (adsorption free energy); *R_L_*, Langmuir dimensionless constant (adsorption tendency favorability). Freundlich: *K_F_*, Freundlich constant related to multilayer capacity; *n*, the heterogeneity factor (indicator for adsorption goodness). Temkin: *K_T_*, Temkin isotherm constant; b, Temkin equilibrium binding constant. *R*^2^, coefficient of determination.

**Table 3:** Thermodynamic data Δ*G*°, Δ*H*°, and Δ*S*° of the CV dye adsorbed onto ZFAC.

Initial Dye Concentration (*C*_0_, mg/L)	Temp. (K)	ln *K_d_*	Δ*G*° (kJ/mol)	Δ*H*° (kJ/mol)	Δ*S*° (J/mol·K)	*R* ^2^
50	296	4.606	−11.571	2.531	47.642	0.034
	308	4.960	−12.142			
	318	4.650	−12.619			
100	296	4.575	−11.149	4.392	52.503	0.331
	308	4.499	−11.779			
	318	4.711	−12.304			
150	296	1.527	−3.868	27.440	105.770	0.949
	308	2.110	−5.137			
	318	2.285	−6.195			
200	296	0.351	−0.915	22.946	80.613	0.984
	308	0.783	−1.883			
	318	0.990	−2.689			

Thermodynamic terms: *K_d_*, equilibrium constant; Δ*G*°, Gibbs free energy change; Δ*H*°, enthalpy change; Δ*S*°, entropy change.

**Table 4:** Kinetic parameters of pseudo-first-order (PFO) and pseudo-second-order model (PSO). Conditions: adsorbate intial concentration *C*_0_ = 25, 50, and 100 ppm; *C*_0_ volume = 250 mL; adsorbent dose = 0.1 g; tepmerature = 23 °C; pH = 7.2; shaking speed = 150 rpm.

*C*_0_ (mg/L)	*q_e_*-exp (mg/g)	PFO			PSO			
		*q*_e_ (mg/g)	*k*_1_ (1/min)	*R* ^2^	*q_e_* (mg/g)	*k*_2_ (g/(mg·min))	*R* ^2^	*h* (mg/(g·min)
25	61.63	54.35	0.2165	0.949	63.69	0.0117	0.972	47.4601
50	121.44	287.01	0.1336	0.795	126.58	0.0012	0.984	19.2270
100	197.14	254.57	0.0781	0.941	212.77	0.0006	0.983	27.1626

**Table 5:** Webber–Moriss kinetic model for intra-particle diffusion (IPD). Results presented for the full experemental data and for the assesed three stages. Conditions: as in Table 4.

*C*_0_	IPD											
	Full-Range			Stage 1			Stage 2			Stage 3		
	*k_id_*	*C*	*R* ^2^	*k_id_*-S1	*C*-S1	*R*^2^-S1	*k_id_*-S2	*C*-S2	*R*^2^-S2	*k_id_*-S3	*C*-S3	*R*^2^-S3
25	14.45	3.534	0.934	18.23	−0.772	0.996	1.82	52.56	0.999	-	-	-
50	14.40	12.911	0.928	22.45	−2.972	0.993	9.18	45.91	0.958	-	-	-
100	21.09	27.166	0.919	35.13	−0.665	0.998	22.11	36.39	0.958	2.48	172.68	0.831

**Table 6:** Comparison of the ZFAC performance with various reported adsorbents on removal of CV dye from polluted water.

Adsorbent	*Q_m_* (mg/g)	Adsorption Conditions				Ref.
		*C*_0_ (mg/L)	T. (°C)	pH	Dose (g/L)	
Magnetically modified activated carbon	44.7	5	20	7	0.5	[58]
Magnetic carbon iron oxide nanocomposite	81.7	240	50	8.5	5.0	[59]
Magnetic charcoal	28.0	40	30	8	1.0	[60]
Zr_3_O/Activated carbon	155.1	100	24	-	0.6	[61]
Nanomagnetic iron oxide	12.7	5	20	7	0.5	[58]
Merck-activated carbon	84.1	80	25	7	1.0	[62]
ZnCl_2_-activated tomato waste carbon	51.6	-	20	8.0	2.0	[50]
ZnCl_2_-activated rice husk carbon	61.6	-	25	10.8	2.0	[63]
Polypyrrole-based activated carbon (PACK)	380.2	100	25	6.9	0.8	[21]
ZFAC	208.3	100	23	7.2	0.6	This work

## Data Availability

Data that support the findings of this study are included within the article.
